# The need for vitamin D assay standardisation in research

**DOI:** 10.1017/S1368980020003328

**Published:** 2020-12

**Authors:** Allison M Hodge

**Affiliations:** 1 Cancer Epidemiology Division, Cancer Council Victoria, 615 St Kilda Rd, Melbourne, VIC, Australia; 2 Centre for Epidemiology and Biostatistics, Melbourne School of Population and Global Health, University of Melbourne, Parkville, VIC, Australia Email allison.hodge@cancervic.org.au

In our recent vitamin D issue (Volume 23 – Issue 7 – May 2020), we have published eleven research papers, one short commentary, a letter to the editor, two invited commentaries, one commentary and an editorial on various aspects of vitamin D as it relates to public health, indicating the level of interest in this topic.

The papers included identified potential associations between low vitamin D and a range of health outcomes, adding to many existing publications on the potential benefits of vitamin D across a range of conditions, for example a recent umbrella review^(1)^. The possibility of vitamin D being beneficial against COVID 19 has even been raised but is only hypothetical at present^(2)^. However, as described in the editorial, there is a lack of agreement between countries and other non-government medical societies and organisations as to the serum concentration of total 25 hydroxyvitamin D (25(OH)D) that reflects inadequacy, and whether concentrations can be further classified as insufficient, sufficient or toxic^(3)^.

As explained in great detail by Sempos and Brinkley^(3)^, a major contributor to the lack of consensus around adequate serum levels of 25(OH)D is the variability in the assays used to measure it. To overcome this and get consensus in studies looking at vitamin D and different outcomes, as well as cut-offs for adequate and inadequate levels of serum 25(OH)D, Sempos and Brinkley^(3)^ are calling for standardisation of vitamin D assays globally. As an example of the difference this can make, a large study across fourteen populations in Europe applied Vitamin D Standardization Program protocols to existing data to re-estimate the prevalence of low 25(OH)D^(4)^. In a population representative sample of adults from Germany, the prevalence of deficiency, defined as < 30 nmol/l, decreased from 25·9 to 15·2%; while in Ireland, it increased from 6·6 to12·3%. This was equivalent to making 10·4 million Germans no longer deficient and finding an extra 267 000 deficient adults in Ireland. Elsewhere, Jakab et al^(5)^ retrospectively standardised the results for 206 samples in the HunMen cohort. The mean total 25(OH)D changed from 53 to 62 nmol/L, and the prevalence of hypovitaminosis D (<75 nmol/L) decreased from 84 to 72%.

To ensure that articles published in PHN can contribute to consensus, and where appropriate to defining adequate and other levels of 25(OH)D, we are proposing to introduce a requirement that all papers published in Public Health Nutrition use standardised 25(OH)D levels that are fit for purpose. The Vitamin D Standardization Program has developed two options for retrospective standardisation of existing measures^(6)^. This would not apply to papers that have already been submitted and are undergoing review but will be taken into account for new submissions starting in 2021. The change will also be reflected in updated Instructions to Authors.
